# Comparison of Standard and Novel Signal Analysis Approaches to Obstructive Sleep Apnea Classification

**DOI:** 10.3389/fbioe.2015.00114

**Published:** 2015-08-27

**Authors:** Aoife Roebuck, Gari D. Clifford

**Affiliations:** ^1^Department of Engineering Science, University of Oxford, Oxford, UK; ^2^The Sleep & Circadian Neuroscience Institute, University of Oxford, Oxford, UK; ^3^Department of Biomedical Informatics, Emory University, Atlanta, GA, USA; ^4^Department of Biomedical Engineering, Georgia Institute of Technology, Atlanta, GA, USA

**Keywords:** audio, MSE, OSA, LPC, MFCCs

## Abstract

Obstructive sleep apnea (OSA) is a disorder characterized by repeated pauses in breathing during sleep, which leads to deoxygenation and voiced chokes at the end of each episode. OSA is associated by daytime sleepiness and an increased risk of serious conditions such as cardiovascular disease, diabetes, and stroke. Between 2 and 7% of the adult population globally has OSA, but it is estimated that up to 90% of those are undiagnosed and untreated. Diagnosis of OSA requires expensive and cumbersome screening. Audio offers a potential non-contact alternative, particularly with the ubiquity of excellent signal processing on every phone. Previous studies have focused on the classification of snoring and apneic chokes. However, such approaches require accurate identification of events. This leads to limited accuracy and small study populations. In this work, we propose an alternative approach which uses multiscale entropy (MSE) coefficients presented to a classifier to identify disorder in vocal patterns indicative of sleep apnea. A database of 858 patients was used, the largest reported in this domain. Apneic choke, snore, and noise events encoded with speech analysis features were input into a linear classifier. Coefficients of MSE derived from the first 4 h of each recording were used to train and test a random forest to classify patients as apneic or not. Standard speech analysis approaches for event classification achieved an out-of-sample accuracy (Ac) of 76.9% with a sensitivity (Se) of 29.2% and a specificity (Sp) of 88.7% but high variance. For OSA severity classification, MSE provided an out-of-sample Ac of 79.9%, Se of 66.0%, and Sp = 88.8%. Including demographic information improved the MSE-based classification performance to Ac = 80.5%, Se = 69.2%, and Sp = 87.9%. These results indicate that audio recordings could be used in screening for OSA, but are generally under-sensitive.

## 1 Introduction

Obstructive Sleep Apnea (OSA) is a disorder that causes breathing to be interrupted repeatedly during sleep. An interruption in breathing results in deoxygenation, leading to a brief arousal (a period of light sleep or wakefulness which is usually not noticed by the individual). Repeated arousals lead to short-term problems, such as daytime sleepiness and poor concentration. OSA is also associated with increased risk of serious chronic conditions, including cardiovascular disease (Monahan and Redline, 2011) and diabetes (Rosenfeld, 2014).

OSA is relatively common, affecting 2–7% of the global adult population; the prevalence is similar in the developed and developing world, with a high variance across ethnic groups (Young et al., 1993; Bearpark et al., 1995; Ip et al., 2001, 2004; Kim et al., 2004; Udwadia et al., 2004; Sharma et al., 2006; Lam et al., 2007). It is usually diagnosed on the basis of an overnight sleep study, where data including photoplethysmography (PPG), respiratory effort, electrocardiography, audio, and activity are typically recorded (Roebuck et al., 2014). Because of the amount of data that are collected and analyzed, the screening process can be expensive, typically costing more than $1000 (Epstein and Dorlac, 1998). In addition, the screening process removes patients from their normal sleeping environment, preventing repeatable unbiased studies. It is estimated that up to 90% of individuals with OSA are undiagnosed and untreated (Young et al., 1997). Screening of OSA is particularly poor in developing countries, where the resources required for conventional screening and diagnosis are often unavailable. While conventional screening of OSA is expensive, some treatment for those diagnosed with the condition can be relatively cheap; oral appliances, which attempt to enlarge the pharyngeal airway during sleep by holding the tongue or jaw forward, are available for as little as £35 (Somno-Guard 3, Tomed, Bensheim, Germany). Lifestyle changes such as losing weight can often resolve mild to moderate OSA, and oral appliances are available for more serious cases. A cheap method of screening for OSA could therefore greatly reduce the burden of OSA on the healthcare system, particularly in developing countries where sleep lab facilities are very limited.

Since audio recordings are particularly easy to collect, and most mobile phones have in-built high quality audio recording capabilities, this article explores whether audio analysis alone is sufficient to diagnose OSA. This is a comparison between two contrasting methods; a standard approach from speech analysis, which has been done before, and a novel approach which captures non-stationary periodicities. There are a number of approaches that are used to determine whether an event is apneic or not, or whether a subject suffers from OSA. Events can be analyzed using LPC (Ng et al., 2008a), mel-frequency cepstrum coefficients (MFCCs) (Cavusoglu et al., 2007), power spectrum (Ng et al., 2008b), energy distribution (Jones et al., 2005, 2006a,b; Cavusoglu et al., 2007), and pitch (Abeyratne et al., 2005). Active devices (Michaelson et al., 2006) and using audio recorded while the subject is awake (Goldshtein et al., 2011) are both methods used to determine whether a subject has apnea or not. Although event detection is more common, it is also more laborious as there needs to be a gold standard for comparison. This involves annotating the entire night’s recording for each subject in the data set. Active devices and using speech signals allow for the subject to be diagnosed rather than identifying individual events. LPC and MFCCs were chosen as the standard approach from speech analysis because they are two of the most common methods used to determine whether an event is apneic or not. From the literature, it can be seen that LPC classifies apneic snores from benign snores with Se = 88% and Sp = 82% for LPC (Ng et al., 2008a); while MFCCs achieve Se = 82% (Cavusoglu et al., 2007). LPC can provide accurate estimates of speech parameters while not being computationally intensive (Rabiner and Schafer, 2007). However, it does assume stationarity, which may not be true over the event duration. MFCCs were used (where the frequency bands are equally spaced on the mel scale) as they approximate the human auditory system’s response more closely than the linearly spaced frequency bands used in the normal cepstrum. This frequency warping can allow for better representation of sound.

## 2 Materials and Methods

### 2.1 Data

The data used in this study were provided retrospectively by collaborators at the Respiratory Medicine Group at the Churchill Hospital (Oxford, UK)^1^. Each subject used a portable home sleep study device, Grey Flash (Stowood Scientific Instruments Ltd., Oxford, UK), which recorded a finger PPG from which oxygen saturation and pulse rate were derived, nasal airflow and nasal sound from a nasal cannula, body movement and body position from an accelerometer, and audio from a microphone placed on the nasal cannula. It should be noted that the subject was in charge of connecting themselves to the device, and that each device was calibrated to have approximately constant gain for the audio signal.

A total of 1354 overnight recordings with associated diagnoses were reviewed, of which only 858 were used in this study; 496 recordings were excluded for being too short (less than 4 h) or for having a diagnosis unrelated to OSA, such as lung disease and asthma. The demographics for the subjects can be found in Table 1, broken down by severity of OSA. The table also shows the apnea hypopnea Index (AHI), the oxygen desaturation index (ODI), and the Epworth sleepiness score (ESS). The AHI is the average number of apneas (cessations of breathing) and hypopneas (episodes of shallow breathing) per hour. The ODI is the average number of oxygen desaturations per hour [where a desaturation is defined as at least a 4% decrease below the previous high; no desaturation can be scored unless the saturation subsequently rises by more than 3% (Stradling and Crosby, 1990)]. The AHI and ODI were automatically calculated by the software used to analyze the data in the clinic (Visi-Download, Stowood Scientific Instruments Ltd., Oxford, UK). ODI definition given above is used by Visi-Download to calculate the ODI. The ESS was derived from a standardized questionnaire designed to measure daytime sleepiness (Johns, 1991).

**Table 1:** **Subject demographics for each sub-group: normal, snorer, mild OSA, moderate OSA, and severe OSA (mean ± σ)**.

Group	Normal	Snorer	Mild	Moderate	Severe
Gender	80 m, 75 f	166 m, 91 f	79 m, 28 f	94 m, 30 f	167 m, 48 f
Age (years)	45.9 ± 17.1	46.5 ± 12.0	50.5 ± 11.4	53.1 ± 12.4	52.5 ± 12.6
Neck (cm)	39.4 ± 4.6	41.4 ± 4.3	41.9 ± 4.1	42.9 ± 3.8	45.0 ± 4.8
Height (cm)	171.2 ± 10.7	173.5 ± 10.4	174.2 ± 9.9	173.0 ± 9.7	175.0 ± 9.1
Weight (kg)	77.7 ± 23.0	96.0 ± 24.2	212.0 ± 48.8	221.2 ± 49.5	247.3 ± 74.4
AHI (events/h)	4.4 ± 7.5	6.4 ± 7.4	10.6 ± 9.0	21.5 ± 11.6	47.5 ± 24.5
ODI (events/h)	3.7 ± 3.5	6.0 ± 5.2	10.3 ± 7.0	22.0 ± 11.6	56.8 ± 32.4
BMI (kg/m^2^)	29.6 ± 7.9	32.0 ± 8.4	31.9 ± 7.9	33.8 ± 8.5	36.9 ± 11.2
ESS	11.0 ± 5.6	12.0 ± 5.2	12.2 ± 4.7	12.7 ± 4.7	14.1 ± 5.3

*Neck, neck circumference; m, male; f, female*.

### 2.2 Annotation and segmentation of data

Twenty-two subjects had specific events identified and labeled using the Visi-Download software; their demographics can be found in Table 2. These subjects were chosen because they were the first subjects that were collected that met the selection criteria, i.e., they were diagnosed as normal, snorer, mild/moderate/severe OSA, and the recordings were longer than 4.5 h with all signals present. The labeling of events followed a protocol that involved dragging an event marker across the relevant section of data. The annotations were made by a clinical research fellow, with 2 years of experience in sleep medicine. Each event was labeled with one of three classes: F, first breath after apnea (or choke); S, snoring; and N, noise events. Only 22 records were annotated due to the time and financial constraints (to annotate a single record took at least one hour). A total of 175 choke/first breath (F) events, 201 snoring (S) events, and 190 noise (N) events were annotated. The entire event, regardless of duration, was annotated.

**Table 2:** **Demographics of annotated subjects (mean ± σ), m, male; f, female**.

Parameter	Subjects (mean ± σ)
Gender	17 m, 5 f
Age (years)	48.9 ± 15.3
Neck (cm)	45.7 ± 3.8
Height (cm)	177.3 ± 10.7
Weight (kg)	107.4 ± 24.4
AHI (events/h)	32.4 ± 31.6
ODI (events/h)	35.7 ± 34.5
BMI (kg/m^2^)	34.3 ± 8.9
ESS	11.7 ± 5.3

### 2.3 Methods

The analysis is separated into two approaches. First, linear discriminant analysis (LDA) was used to build a classifier to differentiate between choke/first breath events (F) and snoring or noise events (S/N) using standard features taken in the literature (linear predictive coding (LPC) and cepstral coefficient analysis). Detection of candidate events was performed by hand. These two methods were chosen as they are standard state of the art, and can be used as a baseline comparison. Then, a novel approach for feature extraction (at least in audio analysis) which requires no segmentation of data, multiscale entropy (MSE), was applied to the raw audio. Both LDA and a random forest (RF) were then trained to classify patients as either requiring treatment or not. Before describing the experimental set-up, a brief overview of the techniques is given.

#### 2.3.1 Linear Predictive Coding

Linear predictive coding is a commonly used speech analysis technique as it provides an accurate representation of speech. Specification of the parameters of the linear predictor is a wide topic and a large number of other approaches have been proposed. In fact, the autocorrelation method is the most common (Rabiner and Schafer, 2007) and it is used, for example, for speech coding in the GSM standard. Full details can be found in Chapter 6 of Rabiner and Schafer (2007).

#### 2.3.2 Cepstral Analysis

Bogert et al. (1963) defined the *cepstrum* as the inverse Fourier transform (IFT) of the log magnitude spectrum of a signal. Cepstral analysis makes use of the source-filter model of speech production. It is widely used in speech processing, particularly for pitch estimation (Rabiner and Schafer, 2007). The cepstrum is created by taking the IFT of the logarithm of the estimated spectrum of a signal. There is a complex cepstrum, a real cepstrum, a power cepstrum, and phase cepstrum. The power cepstrum is often used in the analysis of human speech. Weighted cepstrum distance measures have a direct equivalent interpretation in terms of distance in the frequency domain. This is important in models for human perception of sound which are based on frequency analysis carried out in the inner ear (Rabiner and Schafer, 2007). Davis and Mermelstein (1980) used this fact as the basis of the MFCCs. The idea behind MFCCs is to compute a frequency analysis based on a filter bank with approximately critical band spacing of the filters and bandwidths. The frequency bands are equally spaced on the mel scale, which approximates the human auditory system’s response better than the normal cepstrum. See Chapter 5 of Rabiner and Schafer (2007) for more details.

#### 2.3.3 Multiscale Entropy

Multiscale entropy is a method of measuring the complexity of a finite length time series (Goldberger et al., 2000; Costa et al., 2003; Vrhovec, 2009). MSE has been applied to heart rate and movement data, which has similar issues to the audio signal, such as non-stationarity. Costa et al. (2002) noted that traditional algorithms indicated that certain pathological processes had a higher complexity than healthy dynamics with long-range correlations. The authors suggested that this paradox was due to the fact that conventional algorithms fail to account for the multiple time scales inherent in healthy physiological dynamics. Due to this hypothesis, MSE was developed and was found to robustly separate healthy and pathological groups. Our motivation for using the technique here is that it may catch the low entropy at short time scales (a few minutes) and the fractured sleep (and hence higher entropy at longer time scales – of the order of hours) inherent in OSA.

MSE is calculated as follows: given an *N*-point time series {*x*_1_, …, *x_i_*, …, *x_N_*}, a consecutive coarse-grained time series can be constructed by averaging a successively increasing number of data points in non-overlapping windows.

Each element of the coarse-grained time series, *y_j_*^(*τ*)^, is calculated according to the equation: $y_{j}^{\left(\tau \right)}=\frac{1}{\tau }\sum_{i=(j-1)\tau +1}^{j\tau }x_{i}$ where *τ* represents the scale factor and {1 ≤ *j* ≤ *N/τ*}. The length of each coarse-grained time series is *N/τ*. For scale *τ* = 1, the coarse-grained time series is simply the original time series. The sample entropy (ℋ_s_) is then calculated for each of the time series and can be plotted as a function of the scale factor. ℋ_s_ quantifies the regularity of a time series and is the negative natural logarithm of the probability that two sequences similar for *m* points remain similar at the next point, where self-matches are not included. Given *N* data points from a time series *x*(*n*) = *x*(1), *x*(2),…, *x*(*N*), the algorithm forms *N* − *m* + 1 vectors *X*(1),…, *X*(*N* − *m* + 1) defined by *X*(*i*) = [*x*(*i*), *x*(*i* + 1),…, *x*(*i* + *m* − 1)], for {1 ≤ *i* ≤ *N* − *m* + 1}. The vectors, *X*, represent *m* consecutive values of the signal, commencing with the *i*th point. The distance between *X*(*i*) and *X*(* j*), *d* = [*X*(*i*), *X*(* j*)], is then calculated as the maximum absolute difference between their respective scalar components as follows: *d*[*X*(*i*), *X*(*j*)] = *max_k_*_= 1,2,…,_ *_m_*(|*x*(*i* + *k*) − *x*(*j* + *k*)|) For a given *X*(*i*), the number of *j*’s {1 ≤ *j* ≤ *N* − *m, i* ≠*j*} are counted, such that the distance between *X*(*i*) and *X*(*j*) is less than or equal to *r* SDs and the following function is calculated: $B_{r}^{m}(i)=\frac{1}{N-m-1}\sum_{j=1,j\neq i}^{N=m}\Theta (r.\sigma -d[X(i),X(j)])$ where Θ is the Heaviside function (Θ (*z* ≥ 0) = 1) and (Θ (*z* ≤ 0) = 1), σ is the standard deviation of the signal *x*(*n*) and *r* is a tolerance window. $B_{r}^{m}$ is calculated as follows: $B_{r}^{m}=\frac{1}{N-m}\sum_{i=1}^{N=m}B_{r}^{m}(i)$. The dimension is then increased to *m* + 1 and $A_{r}^{m}(i)$ is calculated as follows: $A_{r}^{m}(i)=\frac{1}{N-m-1}\sum_{j=1,j\neq i}^{N=m}\text{Θ}(r.\sigma -d[X(i),X(j)])\;A_{r}^{m}$ is then given by: $A_{r}^{m}=\frac{1}{N-m}\sum_{i=1}^{N-m}A_{r}^{m}(i)$ and the sample entropy is given by the negative logarithm of the ratio of $A_{r}^{m}\;\text{to}\;B_{r}^{m}:ℋs(m,r,N)=-\ln\;\left(\frac{A_{r}^{m}}{B_{r}^{m}}\right)$. From these equations, it is clear that ℋ_s_, and hence, MSE is a function of three parameters: *m*, *r* and *N*.

#### 2.3.4 Classification

Two classifiers were compared for the estimation of OSA severity: a simple linear classifier and a RF. These methods were chosen as standard linear and non-linear benchmark classifiers. Since there were less than 1000 labeled events for classifying choke/first breath vs. snore/noise, only a linear approach was used on that data.

##### 2.3.4.1 Linear discriminant analysis

Linear discriminant analysis is a well-known method for drawing a linear boundary between the values of a feature set, and has been used in a variety of applications such as image retrieval and face recognition. Classical LDA projects the data onto a lower-dimensional vector space such that the ratio of the between-class distance to the within-class distance is maximized, thus achieving maximum discrimination. The optimal projection can be computed by applying the eigen-decomposition on the covariance matrices (Ye et al., 2004).

##### 2.3.4.2 Random forest classification

Random forests are a type of ensemble classifier based on decision trees (Breiman, 2001). Decision trees form a predictive model which uses a set of binary rules to calculate a target value. Training data are passed to the decision tree, which builds a model determining which variable to split on at a given node, what the value of the split is, whether to stop or to split again and when to assign a terminal node to a class. When a large number of trees have been generated, they vote for the most popular class. For the *k^th^* tree, a random vector Θ*_k_* is generated, independent of the past random vectors Θ_1_,…,Θ*_k_*_–1_ but with the same distribution. A tree is grown using the training set and Θ*_k_*, resulting in a classifier *h*(**x**, Θ*_k_*) where **x** is an input vector. A RF is a classifier consisting of a collection of tree-structured classifiers *h*(**x**, Θ*_k_*), *k* = 1,… where the Θ*_k_* are independent identically distributed random vectors and each tree casts a unit vote for the most popular class at input **x**.

A tree-based classifier, developed by Johnson et al. (2012) using a Bayesian framework. The algorithm has many advantages, including high overall performance and automatic handling of missing data, outliers, and normalization. Each tree selects a subset of observations via two regression splits. These observations are then given a contribution, equal to a random constant times the observation’s value for a chosen feature plus a random intercept. Furthermore, the tree also assigns a contribution to missing values for this chosen feature based upon a scaled surrogate. The contributions across all trees are summed to provide the contribution for a single “forest,” where a “forest” refers to a group of trees plus an intercept term. The predicted probability output by the forest is the inverse logit of the sum of each tree’s contribution plus the intercept term. The intercept term is set to the logit of the mean observed outcome.

The core of the model is the custom Markov chain Monte Carlo (MCMC) sampler which iteratively optimizes the forest. This sampling process has a user-defined number of iterations and a user-defined number of resets (each reset involves reinitializing the forest and restarting the iterative process). After mapping the training data onto the quantiles of a normal distribution, the forest is initialized to a null model, with no contributions assigned for any observations.

At each iteration, the algorithm selects two trees in the forest and randomizes their structure. That is, it randomly reselects the first two features which the tree uses for splitting, the value at which the tree splits those features, the third feature used for contribution calculation, and the multiplicative and additive constants applied to the third feature. The total forest contribution is then recalculated and a Metropolis-Hastings acceptance step is used to determine if the update is accepted. The Metropolis-Hastings algorithm is a MCMC method^2^ for obtaining a sequence of random samples from a probability distribution for which direct sampling is difficult (Metropolis et al., 1953; Hastings, 1970). If the update is accepted, the two trees are kept in the forest, otherwise they are discarded and the forest remains unchanged. After a set fraction of the total number of iterations to allow the forest to learn the target distribution (20%), known as the burn-in period, the algorithm begins storing forests at a fixed interval, i.e., once every set number of iterations. Once the number of user-defined iterations is reached, the forest is reinitialized as before and the iterative process restarts. Again after the set burn-in period, the forests begin to be saved at a fixed interval. The final result of this algorithm is a set of forests, each of which will contribute to the final model prediction.

### 2.4 Analysis protocol

#### 2.4.1 Event Classification

For LPC and MFCC analysis, an assumption was made that an event detector would be used to find the sections of interest, or that the entire night would be analyzed on an approximately second-to-second basis. Therefore, only a specified amount of time for each event was analyzed, i.e., the first 0.5, 1, 2, or 3 s of an event. These window sizes are sufficient as only snores/chokes/noise events are being analyzed, and not apneas which, by definition, last >10 s. If an event duration was less than the specified window size, it was not included in the analysis. This meant that as the window size increased, less data were analyzed; Table 3 shows the number of each event type at the different window sizes.

**Table 3:** **The number of each event type at the four different window sizes used**.

Window	0.5 s	1 s	2 s	3 s
F	175	175	155	82
S	201	201	201	159
N	190	189	185	167

##### 2.4.1.1 LPC

**Figure 1:**
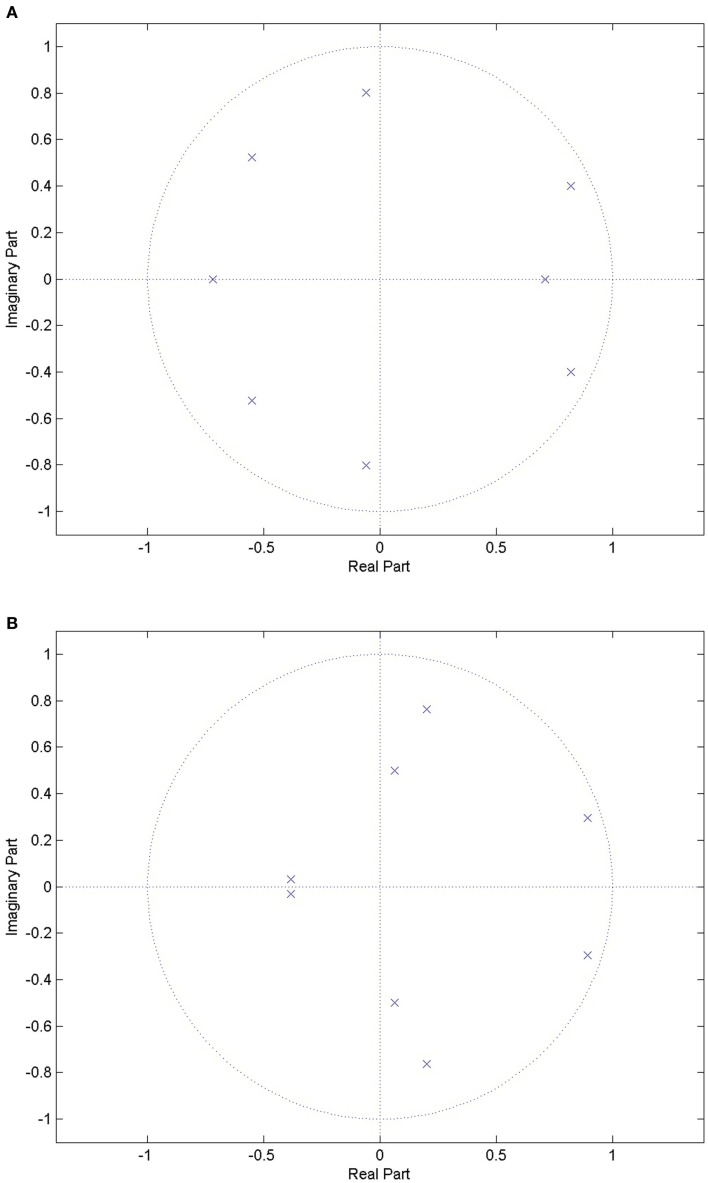
**Pole-zero plots for a choke event and a snoring event**. There are clear differences between the locations of the poles between the two events types, indicating that it might be possible to distinguish between the two. **(A)** Pole-zero plot for a choke event, where the poles are indicated by the blue crosses. **(B)** Pole-zero plot for a snoring event, where the poles are indicated by the blue crosses.

From speech analysis, a general rule of thumb is that, for voiced sounds, two coefficients provide information about each formant frequency. It has been suggested that voiced sounds are identifiable from the first two or three formants (Rabiner and Schafer, 2007). Using a filter order of 8 ensures that the first three formants can be estimated, which is useful in identifying sections of speech in the audio signal. The work of Ng et al. (2008a) looked at the first three formants, and achieved promising results in distinguishing between apneic and non-apneic snoring. Figure 1 shows the pole-zero plots for a choke and a snoring event. There are clear differences between the events, indicating the LPC could be used to distinguish between them.

##### 2.4.1.2 MFCCs

The sampling frequency of the audio data was 4 kHz. Each audio window of interest was detrended and then multiplied by a Hamming window of the same length. A filterbank with 24 filters was used and the entire length of each event was taken to be a single frame, resulting in 12 MFCCs per event.

##### 2.4.1.3 Classification

Five-fold cross-validation was performed. Different combinations of features (LPC, MFCCs, and demographics) were used to fix a boundary between the two classes (F vs. S and N, i.e., identifying apneic sounds from non-apneic sounds). The folds were stratified by subject. Dividing the folds by event would mean that events from a single subject may appear in both data sets, which could lead to a bias in results. For LDA, the discriminant function used fitted a multivariate normal density to each group, with pooled estimates of a diagonal covariance matrix (essentially a naive Bayes classifier).

#### 2.4.2 OSA Severity Classification

##### 2.4.2.1 Feature extraction

Only 240 min of the audio data were analyzed, beginning 30 min into the recording (to remove wakefulness and light sleep at the start of the recording) and ending at 4.5 h. This maximized the number of subjects that could be used, while providing a significant number of events for any given subject. The 4 h window was also recommended by a clinical expert (personal communication; 2010 conversation between Dr. John Stradling and Aoife Roebuck), as OSA will almost certainly manifest within this period if the subject has OSA. The data were preprocessed by taking the variance every 0.5, 1, or 2 s and then the natural logarithm of that time series was taken. This process exaggerated the peaks in the signal, but suppressed large spikes which could overwhelm any metric. Nine MSE coefficients were calculated per subject (*τ* = 1, 2, 4, 8, 16, 32, 65, 130, 180) for *m* = 1: 1: 8 and *r* = 0.1: 0.05: 0.25. The scales chosen attempted to capture the time scales that occur during repeated apneas at both short and long time scales and were fixed per earlier studies (Roebuck and Clifford, 2012; Roebuck, 2014). The values used for *m* and *r* are based on reasonable ranges for physiological data taken from Costa et al. (2003).

##### 2.4.2.2 Classifier training and testing

Again, five-fold cross-validation was carried out on the data. Each time, onefold was held separately to be the test set, while the other four folds were used as the training data set.

The training set was further divided (five times) into training and validation data sets (in the ratio 70:30) in order to find the optimal MSE downsampling rate (*dsr*), *m* value and *r* value. This was performed by a grid search over every possible combination of *dsr*, *m*, *r* and noting the classification accuracy for LDA and the RF separately. The best overall combination was taken to be the one that was chosen most often in the five iterations. Once this combination had been found, the classifier was trained using the entire training set, and tested on the unused test data, for two different feature sets: MSE alone, and MSE plus demographics.

For the RF, 500 trees were used with each tree split on three variables/features. The process was repeated twice with a new seed for 2 × 10^6^ iterations.

## 3 Results

As a comparison, Table 4 shows the performance when using the common clinical thresholds on a variety of features. It is worth noting that both the AHI and ODI are associated with multiple thresholds used for classifying subjects into different categories. Subjects are said to be normal or a snorer if below a threshold of 5 and have mild, moderate, or severe OSA otherwise; 10 or 15 is normal/snorer/mild vs. moderate/severe; 20 or 30 is normal/snorer/mild/moderate vs. severe.

**Table 4:** **Statistics when using clinical thresholds on the demographics, AHI and ODI where both AHI and ODI were automatically calculated by the software**.

Feature	Threshold	Se (%)	Sp (%)	PPV (%)	NPV (%)	Ac (%)
Gender	male	77.5	36.7	45.1	70.8	53.1
Age	50.0	61.7	59.0	50.2	69.6	60.0
Neck	40.0	84.9	40.4	51.7	78.1	59.5
BMI	35.0	45.0	73.8	53.4	66.8	62.3
ESS	15.0	46.4	66.4	48.5	64.5	58.3
AHI	5.0	97.4	55.3	59.2	96.9	72.1
AHI	10.0	92.9	80.1	75.6	94.4	85.2
AHI	**15.0**	**83.5**	**87.6**	**81.7**	**88.8**	**86.0**
AHI	20.0	71.4	94.4	89.5	83.2	85.2
AHI	30.0	53.0	97.9	94.4	75.8	80.0
ODI	5.0	97.6	54.2	58.7	97.2	71.6
ODI	10.0	94.0	81.1	76.8	95.3	86.3
ODI	**15.0**	**85.3**	**90.9**	**86.2**	**90.3**	**88.7**
ODI	20.0	74.3	96.0	92.5	84.9	87.3
ODI	30.0	56.2	98.6	96.4	77.2	81.6

*The metrics in bold are the baseline to beat as this is the classification problem being addressed: normal/snorer/mild OSA vs. moderate OSA/severe OSA*.

The results of the standard speech-based event classification analysis can be found in Table 5, while the results of the MSE analysis using LDA and a RF can be found in Tables 6 and 7 respectively. The best results for each analysis are in bold font in the tables.

**Table 5:** **Performance when using standard speech analysis techniques and LDA on the test data**.

Window	Data	Se (%)	Sp (%)	PPV (%)	NPV (%)	Ac (%)	AUC
0.5 s	L	2.6 ± 3.6	96.3 ± 2.5	12.4 ± 17.0	69.7 ± 8.6	68.0 ± 8.2	0.58 ± 0.13
	C	5.1 ± 5.0	89.7 ± 7.4	17.3 ± 17.4	68.8 ± 9.2	64.1 ± 7.8	0.51 ± 0.10
	L and C	12.6 ± 4.0	81.4 ± 17.3	31.5 ± 17.2	67.0 ± 5.4	60.0 ± 10.7	0.53 ± 0.14
	L and D	29.8 ± 38.9	66.2 ± 28.7	34.6 ± 41.7	71.1 ± 6.2	56.7 ± 12.7	0.57 ± 0.09
	C and D	18.2 ± 20.0	80.2 ± 12.2	29.0 ± 16.8	70.8 ± 12.1	60.7 ± 4.0	0.57 ± 0.14
	L, C, and D	38.8 ± 37.7	69.9 ± 27.3	33.5 ± 31.8	75.6 ± 17.6	57.0 ± 13.5	0.61 ± 0.17
1 s	L	0.9 ± 1.3	97.8 ± 2.2	NaN ± NaN	69.0 ± 9.8	68.0 ± 9.3	0.48 ± 0.07
	C	6.9 ± 6.1	90.6 ± 5.5	21.7 ± 15.9	69.6 ± 11.7	66.1 ± 12.6	0.50 ± 0.08
	L and C	5.6 ± 4.7	86.1 ± 5.6	19.4 ± 19.4	68.7 ± 12.7	62.1 ± 9.0	0.49 ± 0.07
	L and D	26.4 ± 27.9	72.4 ± 22.0	29.7 ± 27.9	69.4 ± 11.2	61.2 ± 14.0	0.53 ± 0.15
	C and D	20.1 ± 11.9	75.4 ± 6.9	26.6 ± 18.3	69.3 ± 2.6	59.2 ± 4.4	0.54 ± 0.15
	L, C, and D	18.5 ± 16.5	80.2 ± 17.8	20.6 ± 15.2	70.1 ± 12.9	63.2 ± 12.9	0.53 ± 0.08
2 s	L	9.1 ± 7.2	93.9 ± 5.8	NaN ± NaN	73.1 ± 16.3	69.8 ± 13.9	0.57 ± 0.06
	C	25.8 ± 12.3	85.6 ± 6.4	42.6 ± 13.7	74.4 ± 4.5	68.6 ± 5.9	0.67 ± 0.08
	L and C	26.0 ± 14.7	86.2 ± 11.7	45.3 ± 15.5	73.7 ± 10.3	67.7 ± 10.2	0.65 ± 0.11
	L and D	31.5 ± 34.8	80.8 ± 20.8	NaN ± NaN	75.6 ± 11.5	64.9 ± 6.9	0.62 ± 0.07
	C and D	31.0 ± 19.2	83.0 ± 11.2	37.5 ± 31.2	76.4 ± 11.2	68.5 ± 8.1	0.65 ± 0.09
	L, C, and D	39.4 ± 19.6	85.0 ± 7.6	48.5 ± 20.5	77.6 ± 10.6	70.6 ± 7.0	0.73 ± 0.03
3 s	L	6.4 ± 6.1	93.9 ± 4.9	25.5 ± 27.7	80.4 ± 7.5	77.0 ± 8.2	0.61 ± 0.08
	C	10.0 ± 13.7	94.5 ± 5.0	NaN ± NaN	81.8 ± 9.3	78.9 ± 10.5	0.62 ± 0.08
	L and C	28.7 ± 25.6	93.6 ± 7.4	45.3 ± 41.0	86.0 ± 13.0	82.1 ± 11.0	0.71 ± 0.19
	L and D	17.3 ± 19.7	86.3 ± 13.0	14.2 ± 8.5	81.0 ± 13.3	73.8 ± 15.4	0.68 ± 0.17
	C and D	34.8 ± 19.1	89.7 ± 10.6	52.6 ± 19.4	84.4 ± 6.5	77.6 ± 4.9	0.76 ± 0.10
	L, C, and D	29.2 ± 17.4	88.7 ± 8.9	49.1 ± 29.9	83.3 ± 9.1	**76.9 *±* 10.6**	**0.73 *±* 0.11**

*L = LPC, C = MFCC, D = demographics. NaN indicates that the classifier never identified a true positive. The metrics in bold indicate the best performance*.

**Table 6:** **LDA Performance when using MSE and demographics on the test data**.

Features	Data set	Se (%)	Sp (%)	PPV (%)	NPV (%)	Ac (%)	AUC
MSE*_aud_*	Train	42.0 ± 14.0	81.8 ± 9.5	62.5 ± 7.2	68.7 ± 2.8	66.2 ± 1.1	0.66 ± 0.01
	Test	41.1 ± 14.3	78.5 ± 11.7	58.8 ± 15.6	67.1 ± 8.2	63.3 ± 5.2	0.64 ± 0.03
MSE*_aud_* + demos	Train	57.0 ± 4.0	78.7 ± 3.3	63.8 ± 1.3	73.7 ± 1.6	70.2 ± 1.3	0.76 ± 0.01
	Test	59.1 ± 7.7	77.5 ± 2.8	64.2 ± 5.8	73.1 ± 7.5	69.6 ± 3.4	0.74 ± 0.03

**Table 7:** **Performance of the RF when using MSE and demographics on the test data**.

Features	Se (%)	Sp (%)	PPV (%)	NPV (%)	Ac (%)	AUC
MSE	66.0 ± 6.8	88.8 ± 1.8	79.0 ± 5.1	80.1 ± 4.1	80.0 ± 3.2	0.86 ± 0.04
MSE + demos	69.2 ± 5.9	87.9 ± 3.9	79.0 ± 5.3	81.2 ± 5.8	**80.5 *±* 4.9**	**0.88 *±* 0.04**

*The metrics in bold indicate the best performance*.

Note that the standard speech analysis techniques have an extremely low sensitivity. Adding demographics to the analysis almost always increases the sensitivity, but only by a moderate amount. Increasing the window size has a moderate effect, but the sensitivity remains low and the variance is high. For the classic speech analysis approach, the best results were consistently obtained when using a combination of LPC, MFCCs, and demographics, for a 3-s window size, achieving Ac = 76.9%, Se = 29.2%, Sp = 88.7%, AUC = 0.73.

As can be seen in Tables 6 and 7, the best performance is obtained when *MSE* + *demos* are used in the analysis; the RF achieved Ac = 80.5% and AUC = 0.88 during validation. However, using *MSE* alone achieves comparable results within the variance over the folds. There is a better balance between Se (66.0%) and Sp (88.8%), unlike in the event classification approach, and the AUC is also higher (0.86 compared to 0.84).

## 4 Discussion

As can be seen in Table 4, demographics or questionnaires alone lead to a poor performance for classifying OSA severity; they are either sensitive or specific, never both, and accuracy is only slightly better than random chance (ranging from 53 to 62%). This is not unexpected, particularly when subjects have been asked to note down these figures themselves rather than being measured and recorded by a healthcare professional; studies have shown that people are poor at self-reporting height and weight (Engstrom et al., 2003). It would therefore be prudent to move away from the use of such information and perhaps rely on objective signals only, if they provide a lower error rate. Both the AHI and ODI are good classification features. It is clear from Table 4 that a threshold of 15 provides the best classification. This separates normal/snorer/mild vs. moderate/severe, and is appropriate for this work as this is the classification problem being addressed. Using this threshold gives performance statistics in the high-80% to low-90% range.

It is clear that the data used for the standard speech-based classifier approach is heterogeneous, and that each fold is significantly different, as evidenced by the large SD values for some of the metrics. This is likely due to the lack of annotations, i.e., there are insufficient data for this analysis or there are too many features. It should be noted that in the training data set, there are up to 30 features (when LPC + MFCCs + demos are combined) but only 56 subjects. This causes the problem to be under-specified, and leads to poor results on the test data. In addition, using three or more annotators would make the annotations more robust. Only those events where two or more annotators agree would be used in the analysis, and could improve the performance of the classifier.

The standard speech-based approach was unable to exceed an Ac of 82% when classifying by subject. Table 5 indicates that using LDA results in a very specific classifier (in the high 90 s), but very low sensitivity (less than 40%). In addition, the PPV could not be computed for some of the combinations of features, because the classifier never identified a true positive.

The fact that the combination of LPC and MFCC consistently provided the best accuracy in determining whether the event was either a choke or noise/snore, regardless of window size, indicates that the LPC coefficients and the MFCCs provided complementary information. The addition of demographics also improved performance. It is worth noting that less data were used at 3 s than at 0.5 s (decreased from 391 noise/snore events, 175 chokes at 0.5 s to 326 noise/snore, 82 chokes at 3 s). The ratio between the classes has completely changed at 3 s.

There are a number of limitations to this approach. Annotating the data is labor intensive and, ideally, there should be three annotators to ensure the quality of the annotation. In this work, one clinical research fellow with 2 years of training labeled the data, which is not optimal.

Assuming that the data were recorded in a low noise environment, the performance achieved is insufficient for screening subjects. Even with the performance metrics as good as they are here, the accuracy needs to be much higher (high 80%) for this approach to be clinically acceptable (Collop et al., 2007), although the ability of portable monitors to correctly diagnose subjects with OSA needs further validation.

The results presented here are lower than those reported in the literature [Se = 88% and Sp = 82% for LPC (Ng et al., 2008a); Se = 82% for MFCCs (Cavusoglu et al., 2007)]. However, many approaches consider in-sample classification which leads to a significant over-estimation of out-of-sample performance. In addition, the literature considers a different classification problem, i.e., thresholding on a given feature to differentiate apneic snores from benign snores, whereas, in the analysis above, the first breath after an apnea has been differentiated from benign snores and noise, which will also influence performance. The poorer performance could be due to the lack of annotations in this analysis, resulting in an under-specified system. In addition, none of the snoring events used in this analysis have been graded. The availability of a grading may have improved performance. It is worth noting that the classification performance is on the classes of sounds themselves, and not for a subject overall.

Finally, the need for an event detector, and then downstream classification to turn the classified events into a diagnosis are likely to degrade the performance further.

In contrast to the speech-based approaches and events classification, the MSE approach yielded a much higher Se, PPV, and AUC with marginal drops in Ac and Sp. Moreover, the addition of demographics made little difference to the classification performance, indicating that the technique may be more suitable as a general tool. The use of an RF applied to MSE coefficients requires very little preprocessing and no segmentation or event detection. While the sensitivity does not surpass 70%, this technique may be suitable as a post-screening test after screening with a pulse oximeter, particularly in settings where there is significant noise from external sources.

## Conflict of Interest Statement

The authors declare that the research was conducted in the absence of any commercial or financial relationships that could be construed as a potential conflict of interest.

## Funding

This research was supported by the RCUK Digital Economy Programme grant # EP/G036861/1 (Oxford Centre for Doctoral Training in Healthcare Innovation) and the University of Oxford Sleep and Circadian Neuroscience Institute (SCNi), Centre Grant # 098461/Z/12/Z.

## Footnotes

^1^This study was approved by the NHS HRC National Research Ethics Service (NRES) South West REC Centre, Bristol, UK (REC reference SW/12/0211).

^2^MCMC methods involve sampling from probability distributions by constructing a Markov chain (a memoryless mathematical system that undergoes transitions from one state to another, among a finite number of possible states) that has the desired distribution as its equilibrium distribution.
